# GPX2 promotes development of bladder cancer with squamous cell differentiation through the control of apoptosis

**DOI:** 10.18632/oncotarget.24627

**Published:** 2018-03-23

**Authors:** Taku Naiki, Aya Naiki-Ito, Keitaro Iida, Toshiki Etani, Hiroyuki Kato, Shugo Suzuki, Yoriko Yamashita, Noriyasu Kawai, Takahiro Yasui, Satoru Takahashi

**Affiliations:** ^1^ Department of Experimental Pathology and Tumor Biology, Nagoya City University, Graduate School of Medical Sciences, Nagoya, Japan; ^2^ Department of Nephro-Urology, Nagoya City University, Graduate School of Medical Sciences, Nagoya, Japan

**Keywords:** GPX2, oxidative stress, bladder cancer, squamous differentiation

## Abstract

Herein, we elucidated the molecular mechanisms and therapeutic potential of glutathione peroxidase 2 (GPX2) in bladder cancer. GPX2 expression gradually increased during progression from normal to papillary or nodular hyperplasia (PNHP) and urothelial carcinoma (UC) in a rat N-butyl-N-(4-hydroxybutyl) nitrosamine (BBN)-induced bladder carcinogenesis model. GPX2 overexpression was more marked in UC with squamous differentiation (SqD) than in pure UC. Clinical intraepithelial lesions of papillary UC and invasive UC with SqD also had strong GPX2 expression in human radical cystectomy specimens. In addition, prognostic analysis using transurethral specimens revealed that low expression level of GPX2 predicted poor prognosis in patients with pure UC. Further, UC cell lines, BC31 and RT4, cultured *in vitro* also overexpressed GPX2. Knock-down of GPX2 induced significant inhibition of intracellular reactive oxygen species (ROS) production, in addition to significant growth inhibition and increased apoptosis with activation of caspase 3 or 7 in both BC31 and RT4 cells. Interestingly, tumor growth of BC31 cells subcutaneously transplanted in nude mice was significantly caused the induction of apoptosis, as well as inhibition of angiogenesis and SqD by GPX2 down-regulation. Our findings demonstrated that GPX2 plays an important role in bladder carcinogenesis through the regulation of apoptosis against intracellular ROS, and may be considered as a novel biomarker or therapeutic target in bladder cancer.

## INTRODUCTION

Bladder cancer is the ninth most common cancer, with an estimated global incidence of 429,000 in 2012 [1], and the development of new treatment modalities is impending based on the clarification of therapeutic molecular mechanisms. Urothelial carcinoma (UC) of the bladder, accounting for about 90% of all bladder tumors, is the second most common urological malignancy after prostate cancer. The most important risk factors for bladder cancer are smoking, aging, and genetic predisposition [2, 3], and solid primary cancers arise via a multi-step process of accumulated genetic alterations [2]. However, the molecular mechanisms of bladder carcinogenesis are not yet clearly elucidated.

Recent evidence suggests that oxidative stress induced by the above-mentioned risk factors is involved in the progression of bladder carcinogenesis. Thus, there is heightened interest in the role of oxidative stress and the status of antioxidant agents in bladder cancer [4–6], as well as antioxidant mechanisms that can be manipulated as therapeutic targets for cancer prevention or as prognostic biomarkers [7–10]. The glutathione redox system is the most important antioxidant agent that protects against intracellular damage induced by oxidative stress in humans [11, 12]. Glutathione peroxidase (GPX), a selenoprotein and a member of the glutathione peroxidase family, is a key enzyme of the glutathione redox system. So far, eight different isoforms of GPX have been reported in mammals [12, 13]. They appear to have antioxidant function at several locations and cellular components. In particular, GPX1 is ubiquitously found in the cytosol and mitochondria of liver, lung kidney, and red blood cells, and GPX2 is mainly expressed in the cytosol of mammary tissue and the gastrointestinal tract, as well as in the human liver [11, 14, 15].

GPX2 is known to reduce hydrogen peroxide and superoxide radical, and is considered to play a major role in the antioxidant defense system, including protection against oxidative damage from risk factors like food consumption, and smoking [11, 16–19]. On the other hand, GPX2 has also been shown to contribute to the progression of malignant tumor [20]. We had previously shown that GPX2 is overexpressed in breast [21], liver [22], and castration-resistant prostate cancers [23], and clarified the molecular mechanisms underlying its regulation in cancer proliferation, in addition to its significance as a therapeutic target in these malignant tumors. In this report, we extended our investigations based on these previous findings, and elucidated the role and prognostic significance of GPX2 in bladder cancer.

## RESULTS

### GPX2 expression in a rat model of bladder carcinogenesis and in human radical cystectomy specimens

**Figure 1:**
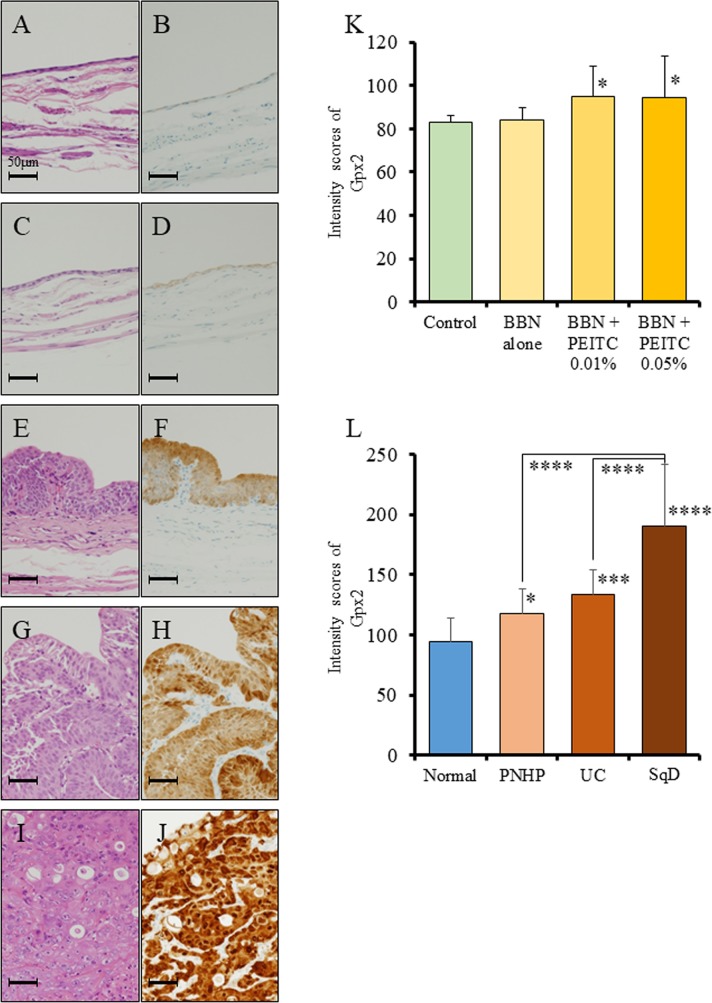
Analysis of Gpx2 expression by immunohistochemistry using a rat bladder carcinogenesis model **(A-D)** HE staining (A, C) and representative immunohistochemistry for Gpx2 (B, D) in normal bladder epithelium obtained from the non-treated group (A, B) and BBN + 0.05% PEITC-treated (C, D) rats. **(E, F)** HE staining (E) and representative immunohistochemistry for Gpx2 (F) in PNHP of the bladder obtained from BBN + 0.05% PEITC-treated rats. **(G, H)** HE staining (G) and representative immunohistochemistry for Gpx2 (H) in papillary pure UC of the bladder obtained from BBN + 0.05% PEITC-treated rats. **(I, J)** HE staining (I) and representative immunohistochemistry for Gpx2 (J) in UC with squamous cell differentiation (SqD) of the bladder obtained from BBN + 0.05% PEITC-treated rat. **(K)** Quantification of intensity of Gpx2 expression in normal epithelium. In BBN + PEITC-treated rats, the expression level of Gpx2 was significantly increased as compared to that in the control non-treated group (Control). Mean ± SD; ^*^p<0.05. **(L)** Quantification of intensity of Gpx2 expression in the BBN + 0.05% PEITC-treated group. The expression level of Gpx2 was significantly increased in PNHP, UC, and SCC as compared to normal epithelium. In addition, the expression level of Gpx2 was the highest in UC with SqD. Mean ± SD; ^*^p<0.05, ^***^p<0.001, ^****^p<0.0001. Nuclei were counterstained with hematoxylin.

**Figure 2:**
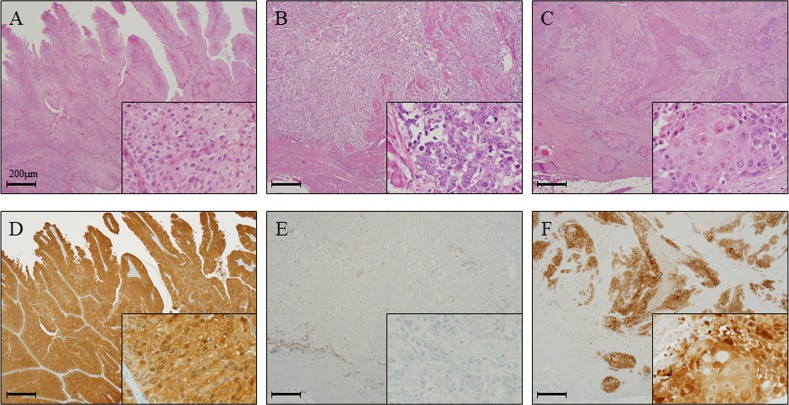
GPX2 expression is elevated in papillary urothelial carcinoma and squamous cell differentiation in radical cystectomy specimens **(A, D)** HE staining (A) and representative immunohistochemistry for GPX2 (D) in papillary UC of the bladder, which showed high expression level similar to the results from the analyses using the animal model. **(B, E)** HE staining (B) and representative immunohistochemistry for GPX2 (E) in advanced invasive UC of the bladder, which showed very low expression level. **(C, F)** HE staining (C) and representative immunohistochemistry for GPX2 (F) in UC with SqD of the bladder, which demonstrated high expression level similar to the results from the analyses using the animal model. Nuclei were counterstained with hematoxylin.

We first examined the status of GPX2, using specimens obtained from a bladder carcinogenesis model induced by treatment of rats with N-butyl-N-(4-hydroxybutyl) nitrosamine (BBN) and 0.05% phenylethyl isothiocyanate (PEITC). We found by immunohistochemical analyses that the protein expression level of Gpx2 in the normal epithelium of the BBN + PEITC-treated group was significantly elevated as compared to that of the non-treated control group (Figure 1A-1D, 1K). Further, the expression level of Gpx2 was significantly higher in papillary or nodular hyperplasia (PNHP) and pure urothelial carcinoma (UC) lesions than in the normal epithelium of BBN + PEITC-treated rats. In addition, Gpx2 expression was demonstrated to be highest in UC with squamous differentiation (SqD) (Figure 1E-1J, 1L). However, in the analyses using clinical radical cystectomy specimens, the expression level of GPX2 was variable. In non-invasive papillary UC tumors, its expression was high (Figure 2A, 2D) while it was low in advanced invasive specimens (Figure 2B, 2E). Interestingly, a high level of GPX2 expression was detected in UC with SqD as observed in the animal model (Figure 2C, 2F). These results suggested that GPX2 expression was induced in urinary epithelium in the initiation phase, was down-regulated in the promotion phase, and was involved in SqD of advanced UC.

### GPX2 expression and prognostic analysis of pure UC patients using transurethral resection (TUR) specimens

**Figure 3:**
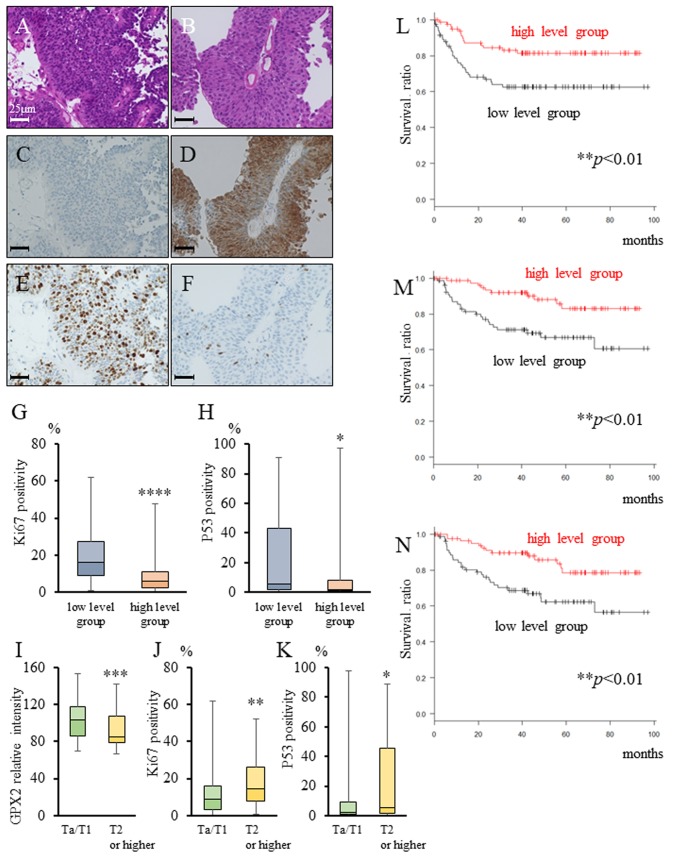
GPX2 and Ki67 expression, and prognostic analysis in transurethral resection (TUR) specimens **(A-F)** HE staining (A, B) and representative immunohistochemistry for GPX2 (C, D) and Ki67 (E, F) In TUR specimens. **(G, H)** Ki67 positivity (G) and P53 positivity (H) in the low (average intensity score < 96.7) and high (average intensity score ≥ 96.7) GPX2 expression groups according to the expression in TUR specimens. ^*^p <0.05, ^****^p <0.001 **(I-K)** Comparison of GPX2 expression score (I), Ki67 positivity (J), and P53 positivity (K) in TUR specimens between cases of stage Ta/T1, and T2 or higher. ^*^p <0.05, ^**^p <0.01, ^***^p <0.001 **(L-N)** Progression-free survival (L), cancer-specific survival (M), and overall survival (N) in patients between the low (n=83), and high (n=86) GPX2 expression groups. ^**^p<0.01.

In both the animal model and human radical cystectomy specimens, GPX2 expression was up-regulated in pure UC. Therefore, immunohistochemical and prognostic analyses were performed using 169 TUR specimens. The clinical course of these patients with bladder cancer is listed in Table 1. We found that the expression of GPX2 was significantly higher in non-invasive bladder cancer patients than in invasive patients, and was inversely correlated with the Ki67 index and P53 positivity (Table 1, Figure 3A-3H). Further, pathological T2 or higher stage patients had significantly lower GPX2 expression as well as higher Ki67 index and P53 positivity than Ta/T1 stage patients (Figure 3I-3K). In addition, the patients with a high level of GPX2 expression in their TUR specimen showed significantly better progression-free survival (PFS), cancer-specific survival (CSS), and overall survival (OS) than those with low level of GPX2 expression (Figure 3L-3N). Table 2 summarizes the prognostic factors for PFS after TUR as determined by univariate and multivariate analyses. Higher GPX2 expression in the TUR specimen had the strongest association with better prognosis after TUR by both univariate and multivariate analyses.

**Table 1:** Patients’ characteristics

Characteristics		GPX2 expression		*p*-value
		Low level group (n=83)	High level group (n=86)	
Median age, years (range)		72 (37-90)	70 (38-87)	n.s.
Sex	Male (%)	65 (78.3)	63 (73.3)	n.s.
	Female (%)	18 (21.7)	23 (26.7)	
Non-invasive or invasive	Non-invasive (%)	3 (0.6)	21 (24.4)	<0.0001^****^
	Invasive (%)	80 (96.4)	65 (75.6)	
Size of main tumor, mm (range)		21 (7-100)	21 (7-65)	n.s.
Solitary or multiple	Solitary (%)	25 (30.1)	30 (34.9)	n.s.
	Multiple (%)	58 (69.9)	56 (65.1)	

GPX2: Glutathione peroxidase 2, n.s.: not significant.

^****^p < 0.0001 statistically significant.

**Table 2:** Univariate and multivariate analyses of baseline and GPX2 expression parameters, and progression free survival in 169 TUR patients

Parameters	Univariate			Multivariate		
	HR	95% CI	*p* value	HR	95% CI	*p* value
Age, 72 ≤ vs. < 71	1.04	0.57-1.90	0.89	0.92	0.51-1.72	0.83
Gender, male vs. female	0.50	0.27-0.93	0.03^*^	0.53	0.28-1.02	0.06
Urine cytology, positive and suspicious positive vs. negative	4.90	1.52-15.9	0.0079^**^	3.43	0.99-11.9	0.051
Tumor multiplicity, multiple vs. solitary	1.86	0.92-3.78	0.09	1.58	0.76-3.27	0.22
Tumor grade, high vs. low	4.0	0.97-16.5	0.06	1.32	0.28-6.22	0.72
Concomitant of CIS, yes vs. no	0.90	0.12-6.53	0.92	0.66	0.09-4.92	0.69
Expression of GPX2, higher vs. lower	0.41	0.22-0.78	0.0061^**^	0.49	0.25-0.97	0.039^*^

CIS: Carcinoma *in situ*, GPX2: Glutathione peroxidase 2, TUR: Transurethral resection of bladder cancer, HR: Hazards ratio, CI: Confidence interval. ^*^p<0.05, ^**^p<0.01 indicates a significant difference.

### GPX2 expression in bladder cancer cell lines, and *GPX2* siRNA transfection in RT4 cells

**Figure 4:**
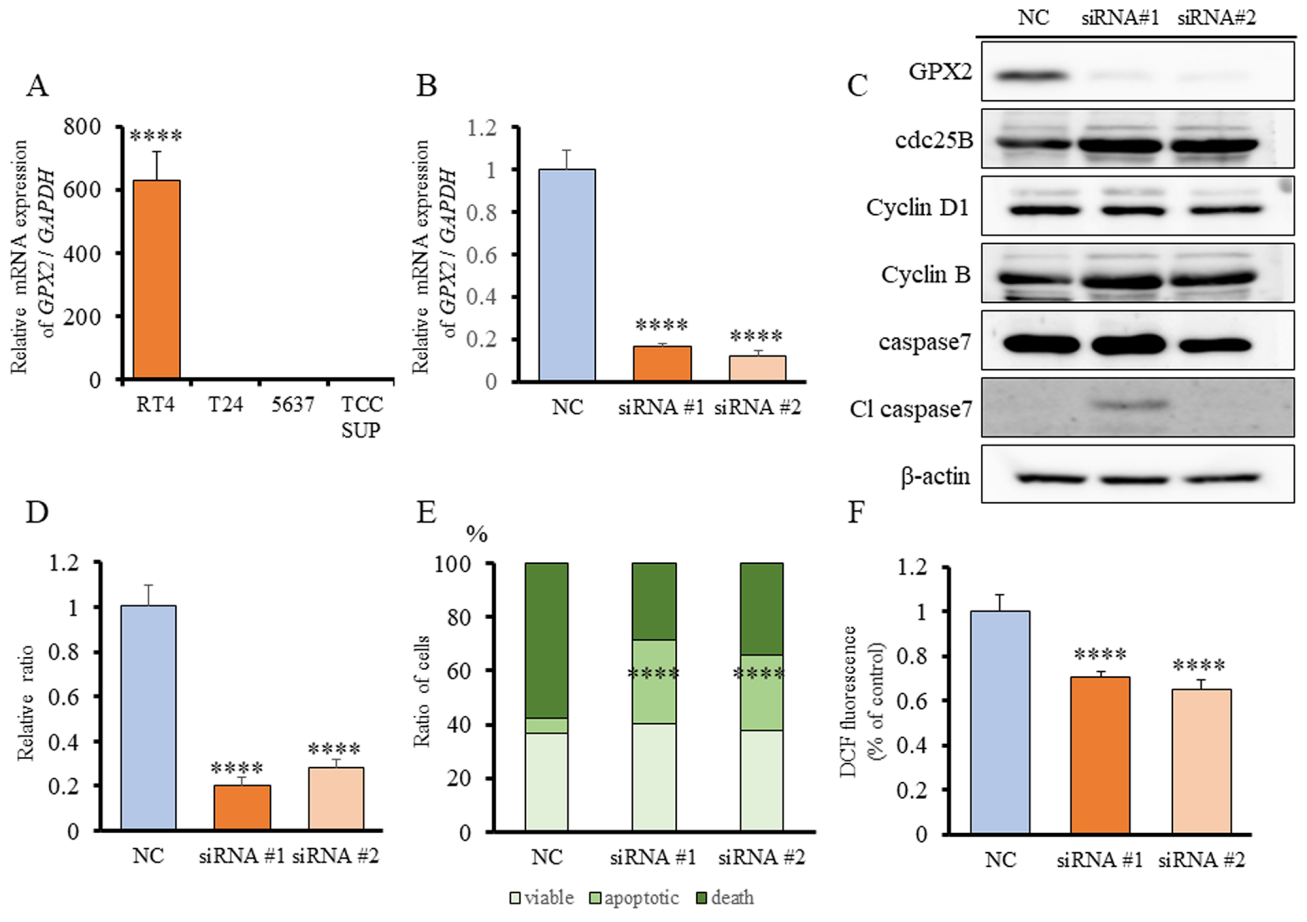
Expression of GPX2 in human bladder cancer cell lines, and *GPX2* siRNA transfection in RT4 cells **(A)** *GPX2* mRNA expression in human bladder cell lines, RT4, T24, 5637, and TCCSUP, was assessed by qRT-PCR. The mRNA expression level of *GPX2* in RT4 cells, established from low grade UC, was significantly higher than in other cell lines established from invasive high grade UC. Mean ± SD; ^****^p<0.0001. **(B)** mRNA expression level of *GPX2* in RT4 cells was confirmed by qRT-PCR 2 days after transfection with two different *GPX2*-targeting and negative control (NC) siRNAs. Mean ± SD; ^****^p<0.0001. **(C)** Western blotting analyses at five days after siRNA transfection of RT4 cells. The expression of GPX2 was reduced, but that of cdc25B, cyclin D1, cyclin B, caspase 7, and caspase 3 were not changed. β-actin was used as internal loading control. **(D)** Proliferation rate of RT4 cells following transfection with *GPX2*-targeting and NC siRNAs. Mean ± SD; ^****^p<0.0001. **(E)** Guava^®^ apoptosis analysis of RT4 cells after siRNA transfection shows that *GPX2* knock-down induced apoptosis. Mean; ^****^p<0.0001. **(F)** DCFH-DA assay was used to quantify intracellular ROS levels after knock-down of *GPX2* by siRNA in RT4 cells. Mean ± SD; ^****^p<0.0001.

To elucidate the mechanisms of tumorigenic ability induced by GPX2, we explored the role of GPX2 on cell proliferation in human UC cell lines, of which RT4 had significantly higher expression of GPX2 than the other UC cell lines, T24, 5637, and TCCSUP (Figure 4A). Therefore, we used RT4 cells for further analyses. Knock-down of GPX2 by two different siRNAs in RT4 was confirmed by quantitative RT-PCR (qRT-PCR) (Figure 4B). Cell proliferation of RT4 cells was significantly suppressed by GPX2 inhibition as compared to the negative control (NC) (Figure 4D). To understand the underlying growth regulatory mechanism by GPX2, we determined whether the levels of proteins associated with cell cycle and apoptosis were altered by inhibition of GPX2 in RT4 cells. Suppression of GPX2 resulted in marked induction of cleaved caspase 7, while no changes in the expression of cell cycle-related proteins were observed (Figure 4C). Therefore, flow cytometry analysis by the Guava^®^ apoptosis assay was performed, and we found that there appeared to be a significant accumulation of apoptotic cells following GPX2 inhibition (Figure 4E). Furthermore, dichloro-dihydro-fluorescein diacetate (DCFH-DA) assay revealed that intracellular ROS level was significantly decreased in the *Gpx2*-siRNA transfected group as compared to the NC (Figure 4F). These results suggested that GPX2 is involved in the maintenance of cell proliferation by protection against caspase-dependent apoptosis via ROS regulation in RT4 cells.

### *Gpx2* siRNA transfection and ROS signals in BC31 cells

**Figure 5:**
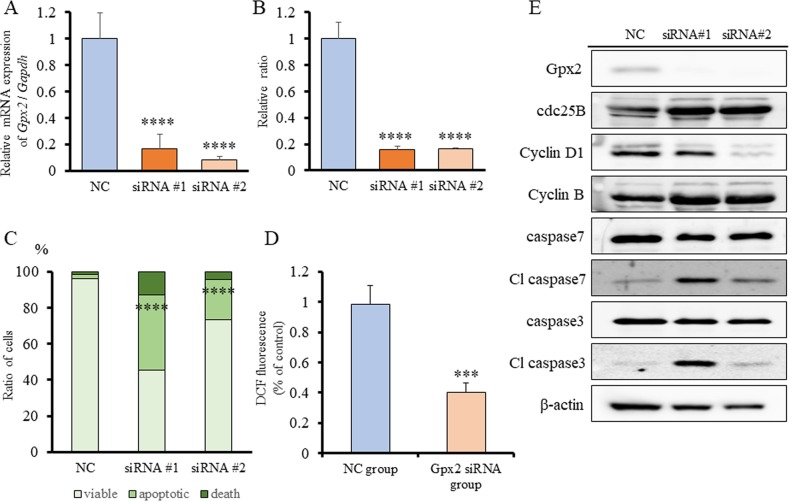
*Gpx2* siRNA transfection and ROS signals in BC31 cells **(A)** mRNA expression level of *Gpx2* in BC31 cells was confirmed by qRT-PCR 2 days after transfection with two different *Gpx2*-targeting and negative control (NC) siRNAs. Mean ± SD; ^****^p<0.0001. **(B)** Proliferation rate of BC31 cells treated with *Gpx2*-targeting and NC siRNAs at 5 days after siRNA transfection. Mean ± SD; ^****^p<0.0001. **(C)** Guava^®^ apoptosis analysis of BC31 cells after siRNA transfection shows that *Gpx2* knock-down induced apoptosis. Mean; ^****^p<0.0001. **(D)** DCFH-DA assay was used to quantify intracellular ROS levels after knock-down of *Gpx2* by siRNA in BC31 cells. Mean ± SD; ^***^p<0.001. **(E)** Western blotting analyses at 5 days after siRNA transfection of BC31 cells. The expression of Gpx2 was reduced, but that of cdc25B, cyclin D1, cyclin B, caspase 7, and caspase 3 were not changed. β-actin was used as internal loading control.

In our previous study, we demonstrated that GPX2 promotes cell proliferation by control of oxidative stress using GPX2 knock-down analyses. To examine the role of GPX2 on cell proliferation and oxidative stress in UC, BC31 cells, which is a rat UC cell line with squamous characterization [24, 25], was used. qRT-PCR analysis revealed that *Gpx2* mRNA levels were inhibited following transfection with two different siRNAs for 2 days (Figure 5A). Similar to RT4 cells, cell proliferation of BC31 cells was significantly decreased by inhibition of GPX2 as compared to NC (Figure 5B). In addition, Gpx2 silencing induced a significant increase in apoptosis with activation of caspases 3 and 7 by western blotting and flow cytometry (Figure 5C, 5E). Further, DCFH-DA assay also revealed that intracellular ROS level was significantly decreased in the *Gpx2*-siRNA transfected group as compared to the NC (Figure 5D). These results suggest that ROS accumulation induced by Gpx2 overexpression is necessary for maintaining cell growth in BC31 cells.

### *In vivo* regulation by Gpx2 of growth of tumors with squamous cell differentiation derived from BC31 cells

**Figure 6:**
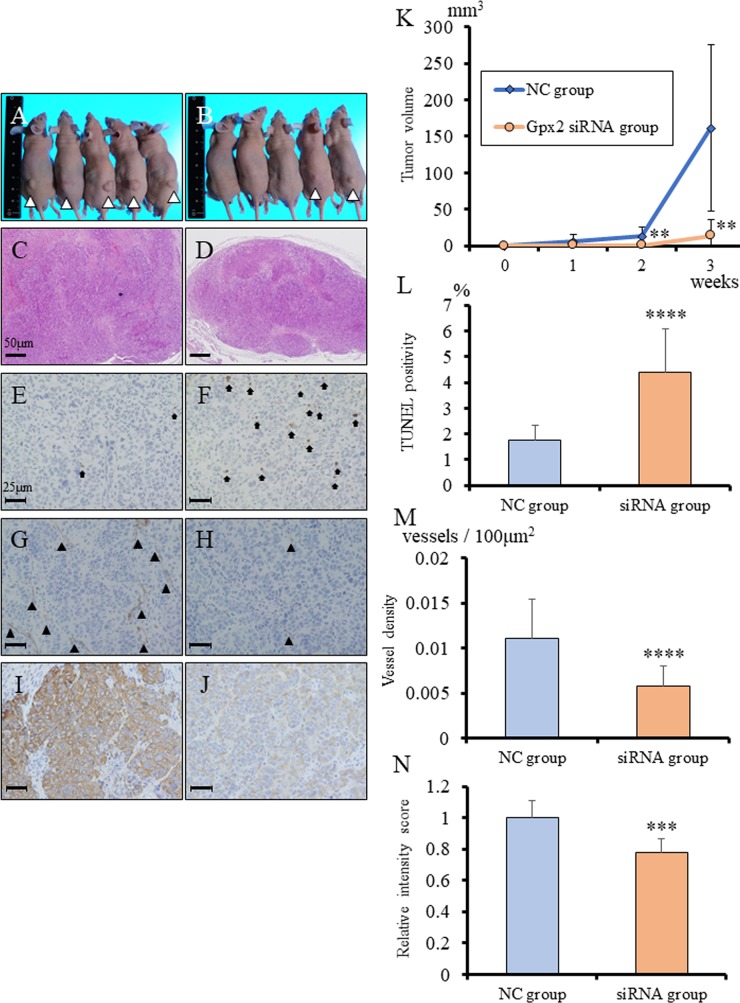
Inhibition of *Gpx2* attenuated subcutaneous BC31 tumor growth, induced apoptosis, and decreased CK14 expression *in vivo* **(A, B)** Morphology of subcutaneous tumors at 3 weeks after transplantation. Tumors derived from BC31 cells transfected with negative control (NC) (A) and *Gpx2*-targeting (B) siRNAs. **(C, D)** HE staining of subcutaneous tumors derived from BC31 cells transfected with *NC*- (C) and *Gpx2*-targeting (D) siRNAs. **(E, F)** Representative TUNEL staining for apoptosis in tumors derived from BC31 cells transfected with NC (E) and *Gpx2*-targeting (F) siRNAs. Black arrows indicate TUNEL-positive cells. **(G, H)** Representative immunohistochemistry for CD31 in tumors derived from BC31 cells transfected with NC (G) and *Gpx2*-targeting (H) siRNAs. Black arrowhead indicates CD31-positive vessel. **(I, J)** Representative immunohistochemistry for CK14 in tumors derived from BC31 cells transfected with NC (I) and *Gpx2*-targeting (J) siRNAs. **(K)** Tumor growth was significantly inhibited in mice following transplantation of BC31 cells transfected with *Gpx2*-targeting siRNA as compared to NC. Mean ± SD; ^**^p<0.01. **(L)** TUNEL assay was performed on tumors derived from BC31 cells transfected with *Gpx2*-targeting and NC siRNAs, and quantified as mean TUNEL labeling percentage based on at least 5 randomly selected high-power microscope fields per individual. Mean ± SD; ^****^p<0.0001. **(M)** Quantification of immunohistochemical intensity of CD31. Positivity was quantified as mean number of vessels/mm^2^ based on at least 5 randomly selected high-power microscope fields per individual. Mean ± SD; ^****^p<0.0001. **(N)** Quantification of immunohistochemical intensity of CK14. Positivity was quantified as mean intensity score based on at least 5 randomly selected high-power microscope fields per individual. Mean ± SD; ^***^p<0.001. Nuclei were counterstained with hematoxylin.

To evaluate the role of GPX2 in growth of UC with SqD, *Gpx2*-siRNA transfected BC31 cells were subcutaneously transplanted in nude mice. Knock-down of *Gpx2* significantly inhibited tumor growth of BC31 cells as compared to the NC (Figure 6A-6D, 6K). To verify the results of the *in vitro* study, the *in vivo* tumor suppressive mechanisms by *Gpx2* attenuation were examined using TUNEL assay. Down-regulation of *Gpx2* significantly increased the proportion of TUNEL-positive apoptotic cells as compared with NC (Figure 6E-6F, 6L). In addition, we examined vascular density in the tumors by immunohistochemistry for CD31. We found that vessel density was significantly decreased in the *Gpx2*-siRNA group than in the NC group (Figure 6G-6H, 6M). We further examined the abundance of cancer cells with squamous differentiation. Immunohistochemical analyses for cytokeratin (CK) 14 revealed that its expression was significantly decreased in *Gpx2*-suppressed tumors as compared to *NC* ones (Figure 6I-6J, 6N). These results suggested that attenuation of Gpx2 suppressed tumor growth of UC with SqD through the induction of apoptosis, as well as inhibition of angiogenesis and squamous differentiation.

## DISCUSSION

To our knowledge, only one previous report has been published regarding the expression level of GPX2 in bladder cancer [26]. However, the stage-dependent expression alteration and detailed molecular mechanisms of GPX2 during bladder carcinogenesis had not been clarified. In the present study, we found that GPX2 expression was elevated by PEITC, which promotes bladder carcinogenesis, in normal epithelium in a two-stage rat urinary bladder carcinogenesis model. In human gastrointestinal tissue, bacterial infection can enhance GPX expression in initiated cells [27], which is thought to be required for antioxidant defense against H_2_O_2_ or other hydroperoxides, resulting in prevention of tumor promotion [17, 28, 29]. Thus, the findings from this study suggest that similar to the mechanisms observed in the gastrointestinal tract, activation of the glutathione redox system in the early stage of carcinogenesis could protect from promotion of bladder cancer development.

To date, the majority of the reports have described GPX2 as an anti-inflammatory and anti-carcinogenic enzyme. Oxidative stress is generally considered to contribute to the promotion of carcinogenesis. Therefore, reduction of cellular oxidation by GPX up-regulation was expected to prevent against tumor promotion. However, several reports including our previous study described that a high GPX2 expression supported the growth of cancer cells by facilitating their proliferation via inhibition of oxidative damage-induced apoptosis [15, 23, 30]. In line with these studies, we also demonstrated here that suppression of GPX2 by siRNA induced as caspase-dependent apoptosis and a decrease in intracellular ROS level in *in vitro* (Figure 4). Our findings suggest that up-regulation of GPX2 is in fact associated with bladder cancer development via its role in regulation of apoptosis and oxidative stress. This is further supported by our study using a rat carcinogenesis model whereby the expression level of Gpx2 was significantly increased in PNHP and pure UC as compared to normal epithelium (Figure 1). These results suggest that once carcinogenic abnormalities had accumulated in normal epithelium, there is progressive increase in expression of GPX2 from PNHP to UC. Overexpression of GPX2 may facilitate uncontrolled cell proliferation, thus, GPX2 appears to provide a selective advantage for pure UC cells and support further malignant growth. Further research is necessary to delineate the upstream regulatory mechanisms that control GPX2 expression.

Recently, the prognostic significance of GPX2 in human malignancies was reported [31–36], and only one report has described that low expression level of GPX2 is associated with poorer prognosis in bladder cancer [26]. Our novel findings demonstrated that GPX2 expression was inversely correlated with both the Ki67 labeling index and the expression of P53, a tumor suppressor gene, that is frequently overexpressed or amplified in invasive higher stage bladder cancer (Figure 3A-3K). Yan and Chen [30] reported that the protective function of GPX2 is P53 dependent, and deficiency of GPX2 promotes susceptibility to oxidative stress-induced apoptosis in MCF7 cells, a human breast cancer cell line. It was also reported that cells with GPX2 knock-down had a higher capability to invade and migrate than the GPX2-expressing controls in colon cancer. Therefore, an increase in P53 in invasive tumors may also bring about decrease in GPX2 expression along with suppression of its protective function. In addition, our results also demonstrated that pure UC patients with low level of GPX2 expression in TUR specimens had significantly poorer PFS, CSS, and OS as compared to patients with high level (Table 1, Figure 3L-3N). From our findings of the role of GPX2 in bladder cancer, we propose that GPX2 may serve as a prognostic biomarker for pure UC patients.

In addition to this, we found in this study that GPX2 was overexpressed in UC with SqD using an animal model, and there were some clinical cases with strong GPX2 expression in UC with SqD using radical cystectomy specimen (Figures 1-2). SqD, defined by the presence of intercellular bridges and/or keratinization, is the most common type of divergent differentiation, occurring in as many as 40% of invasive UC [37, 38]. Limited reports have shown that SqD can predict reduced responsiveness to radiation or chemotherapy, but to date, no report has described the expression level and the role of GPX2 in SqD of UC. It has previously been shown that GPX2 protein is overexpressed in neoplastic transformation of squamous epithelial cells, Barrett’s esophagus, and esophageal squamous cell carcinoma [39]. However, the mechanisms of GPX2 overexpression in those tumors are unclear. Yan and Chen [30] reported that GPX2 expression is regulated by ΔNp63γ, and GPX2 overexpression can inhibit the apoptotic response of cancer cells to oxidative stress. Serewko et al. [39] revealed that GPX2 expression was induced in human oral epithelial cells during squamous cell carcinoma development, among other alterations in genes that were involved in tissue remodeling, life span, as well as growth and differentiation controls. These mechanisms may also contribute to SqD of bladder cancer. In addition, *in vivo* analyses of the BBN-induced rat bladder cancer model following transplantation of BC31 cells with Gpx2 knock-down showed that tumor growth, SqD, angiogenesis were all suppressed, and caused the induction of apoptosis (Figure 6). These results suggested that GPX2 may be an important differentiation biomarker, and a potential target for uncontrollable UC with SqD after radiation or chemotherapy. Further investigation is necessary to validate these findings.

In conclusion, our study demonstrated that GPX2 was enhanced in UC and may play an important role in the proliferation of UC with SqD. Further, higher GPX2 expression was associated with better prognosis in pure UC patients. These findings indicated that GPX2 may be one of the essential biomarkers or therapeutic targets for bladder cancer, and more randomized control trials should be performed in the near future to verify this.

## MATERIALS AND METHODS

### BBN and PEITC-induced bladder carcinogenesis rat model

In a previous study, 6-week-old male F344 rats were given 0.05% BBN in the drinking water for 4 weeks as initiation [40]. Three days after completion of the initiation, a diet containing 0%, 0.01%, or 0.05% PEITC was given for 32 weeks. All bladder tissues obtained from this previous study were reprocessed [40, 41], and neoplastic lesions were reevaluated by experienced pathologists (A. N-I, S. S.).

### Human radical cystectomy specimens

Radical cystectomy specimens were obtained from Nagoya City University Hospital between 2010 and 2014. All specimens were obtained after the patients had provided written informed consent for the use of their tissues, according to an Institutional Review Board-approved protocol, and the approval number was NCU-893. All cases were reevaluated by experienced pathologists.

### Immunohistochemical analyses

Deparaffinized formalin-fixed tissues were incubated with 1:100 diluted anti-GPX2, 1:100 diluted anti-Ki67 (Dako, Glostrup, Denmark), or 1:500 diluted anti-P53 antibody (Leica Biosystems, Wetzlar, Germany) [23], and the rat tissues were also incubated with 1:100 diluted anti-CD31 (Abcam, Cambridge, UK) or 1:100 diluted anti-CK14 (Leica Biosystems) antibody. Antibody binding was visualized by a conventional immunostaining method using an autoimmunostaining apparatus as described previously [21, 23] (HX System, Ventana, Tuscon, AZ).

In the analysis of human TUR specimens, the intensity score of GPX2 expression was evaluated in carcinoma lesions from each patient. In the analysis of the intensity of GPX2 cytoplasmic immunoreactivity during the initiation period in the animal model, the raw intensity data for normal epithelium in each group were compared. Next, the intensity of GPX2 cytoplasmic immunoreactivity in PNHP and UC lesions, which correspond to the promotion period, from the BBN + 0.01% PEITC group was calculated. Further, the intensity of GPX2 cytoplasmic immunoreactivity in PNHP, UC lesions, and UC with SqD lesions from the BBN + 0.05% PEITC group was analyzed. All analyses were performed using the BZ-9000 multifunctional microscope and the associated analysis software (Keyence Japan, Osaka, Japan). The evaluations were repeated 5 times in each patient, and the average intensity score in each lesion was calculated.

### Prognostic analysis of pure UC patients using TUR specimens

Between April 2007 and March 2012, 169 well-characterized patients were newly diagnosed with pure UC originating from the urinary bladder using the initial TUR at Nagoya City University Hospital. The clinical course of these patients with bladder cancer is listed in Table 1. In the analysis of TUR specimens, we calculated the average expression score in each patient, and the median intensity score (96.7) was used as the cutoff to dichotomize the study cohort using the following categories: low level GPX2 expression group (average intensity score < 96.7) and high level GPX2 expression group (average intensity score ≥ 96.7). Two pathologists re-evaluated hematoxylin and eosin (HE) sections of all cases, and the intensity scores of GPX2 immunostaining were evaluated using the Biorevo BZ-9000 microscope and the associated software (KEYENCE, Osaka, Japan).

### Cell culture and treatments

The human bladder cancer cell lines, including a low grade urothelial carcinoma cell line (RT4) [42–46], and high grade urothelial carcinoma cell lines (T24, 5637, and TCCSUP), purchased from American Type Culture Collection (Manassas, VA), and rat bladder cancer with SqD cell line, BC31, which was established from the BBN-induced rat bladder cancer model [24, 25], were cultured as described previously [23]. All experiments were performed in triplicate.

### RNA preparation and qRT-PCR

Total RNA was extracted using Isogen (Nippon Gene, Tokyo, Japan). For the measurement of mRNA level, qRT-PCR was performed as previously described [23]. Primer sequences were 5’-GACACGAGGAAACCGAAGCA-3’ and 5’-GGCCCTTCACAACGTCT-3’ for rat *Gpx2*; 5’-GCCTCCTTAAAGTTGCCATA-3’ and 5’-GCCCAGAGCTTACCCA-3’ for human *GPX2*; and 5’-GCATCCTGCACCACCAACTG-3’ and 5’-GCCTGCTTCACCACCTTCTT-3’ for glyceraldehyde-3-phosphate dehydrogenase (*GAPDH*).

### Western blotting analysis

Cells were lysed in SDS buffer, and 10 μg of protein was resolved in 12% polyacrylamide gels and transferred onto Hybond ECL membranes (GE Healthcare, Piscataway NJ). GPX2, cyclin B (BD Bioscience, Franklin lakes, NJ), as well as cyclin D1, cdc 25B, cleaved caspase 3, caspase 3, caspase 7), and cleaved caspase 7 (Cell Signaling Technology, Beverly, MA) antibodies were used to assess their protein expression levels. Beta-actin expression was evaluated to confirm equal amount of protein loading using a monoclonal anti-beta-actin antibody (Sigma-Aldrich, St. Louis, MO).

### *In vitro* siRNA transfection and cell growth assay

siRNAs targeting rat and human *GPX2* sequences were obtained (Thermo Fischer Scientific, Boston, MA). RT4 (2 × 10^5^) and BC31 (5 × 10^4^) cells were seeded in six-well plates and transfected with 30 nM siRNA using LipofectAMINE RNAiMAX (Thermo Fischer Scientific) according to the manufacturer’s protocol. Non-targeting siRNA with no significant homology to any known rat and human genes was also used as a negative control (NC). Confirmation of GPX2 knock-down was performed on the second day after transfection. For monitoring of cell growth, cells were trypsinized on day 5 after transfection and then counted.

### Flow cytometry analysis

RT4 and BC31 cells (2 × 10^5^) were transfected with GPX2 siRNA for 72 h then cell suspensions were prepared and stained with Guava^®^ ViaCount reagent and propidium iodide according to the Guava^®^ Assay protocol (Guava Technologies, Hayward, CA). Apoptotic analysis and cell cycle phase distributions were determined on a Guava^®^ PCA Instrument using the CytoSoft Software.

### Measurement of intracellular ROS level

For the measurement of intracellular ROS level, the DCFH-DA assay was performed as previously described [23]. The data were normalized by the proliferation rate as measured by the WST-1 assay using the WST-1 Cell Proliferation Reagent (Roche, Basal, Switzerland).

### *In vivo* studies using subcutaneously-injected BC31 cells

Six-week-old male KSN/nu-nu nude mice were obtained from Nippon SLC and maintained as previously described [23]. Twenty-four hours after *Gpx2* siRNA transfection into BC31 cells, they were trypsinized and suspended in serum-free DMEM, after which 1 × 10^5^ cells per mouse were injected subcutaneously into five normal and five castrated mice. NC-siRNA treated cells were also injected as controls. Tumor volume was calculated every week, and three weeks after inoculation, the four groups of mice were sacrificed. Tumor volume was calculated using the formula, V = (A x B^2^) / 2, where V represented volume in mm^3^, and A and B represented the long and the short diameter in mm, respectively.

All animal experiments were performed under protocols approved by the Institutional Animal Care and Use Committee of Nagoya City University School of Medical Sciences, and the approval number was H25M-54.

### TUNEL assay

Apoptotic cells in deparaffinized tissues were detected by the terminal deoxy nucleotidyl transferase-mediated dUTP nick end labeling (TUNEL) assay performed using an *In situ* Apoptosis Detection Kit from Takara (Otsu, Japan) as per the manufacturer’s protocol. Five randomly selected microscopic fields in each group were used to calculate the relative ratio of TUNEL-positive cells.

### Statistical analysis

Differences between the various parameters were assessed using Student’s *t*-test, ANOVA, Tukey’s post-hoc methods, Kruskal-Wallis test, and chi-square test, whichever was appropriate. The PFS, CSS, and overall survival (OS) curves were estimated by the Kaplan–Meier method, and log-rank test was applied to compare survival between groups. To identify prognostic factors for PFS after the TUR, we evaluated seven variables (age, gender, urine cytology before the TUR, tumor multiplicity, tumor grade, concomitant carcinoma *in situ* [CIS] and GPX2 expression level) by univariate and multivariate analyses using the Cox proportional hazard regression model. A p-value < 0.05 was considered to indicate a statistically significant difference. Statistical analyses were performed using the EZR software (Saitama Medical Center, Jichi Medical University, Yakushiji, Japan).
